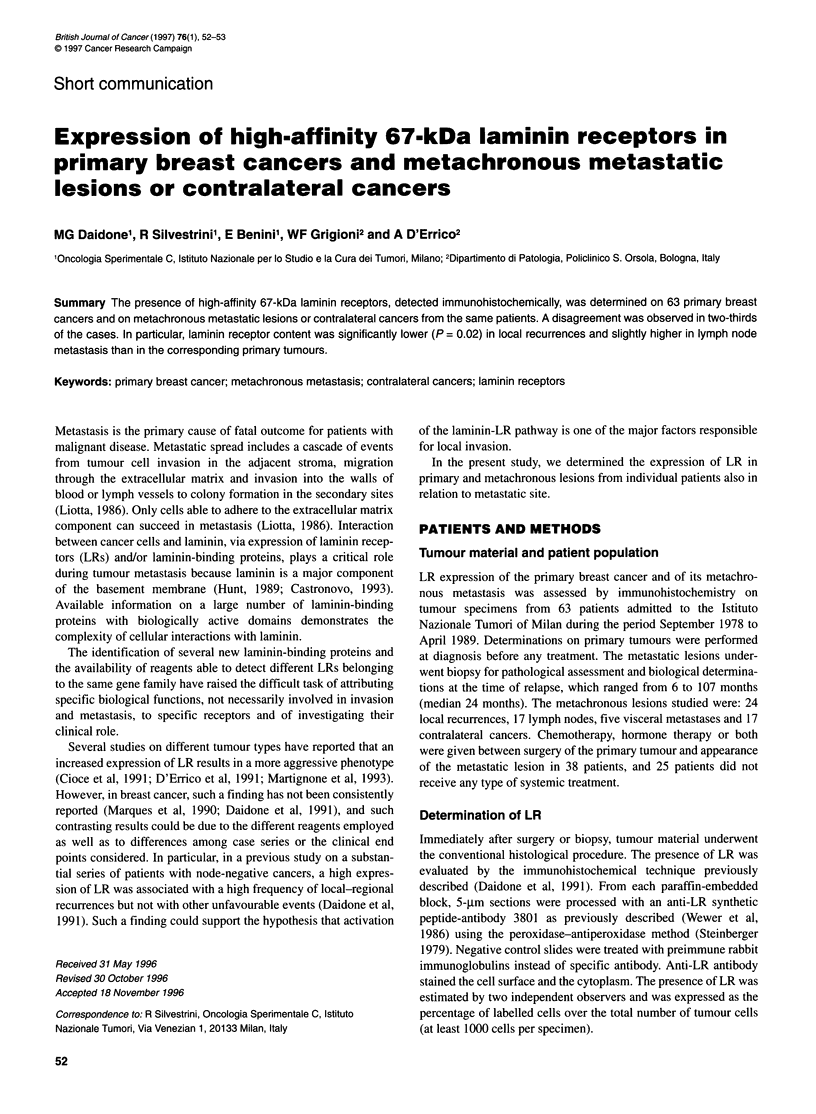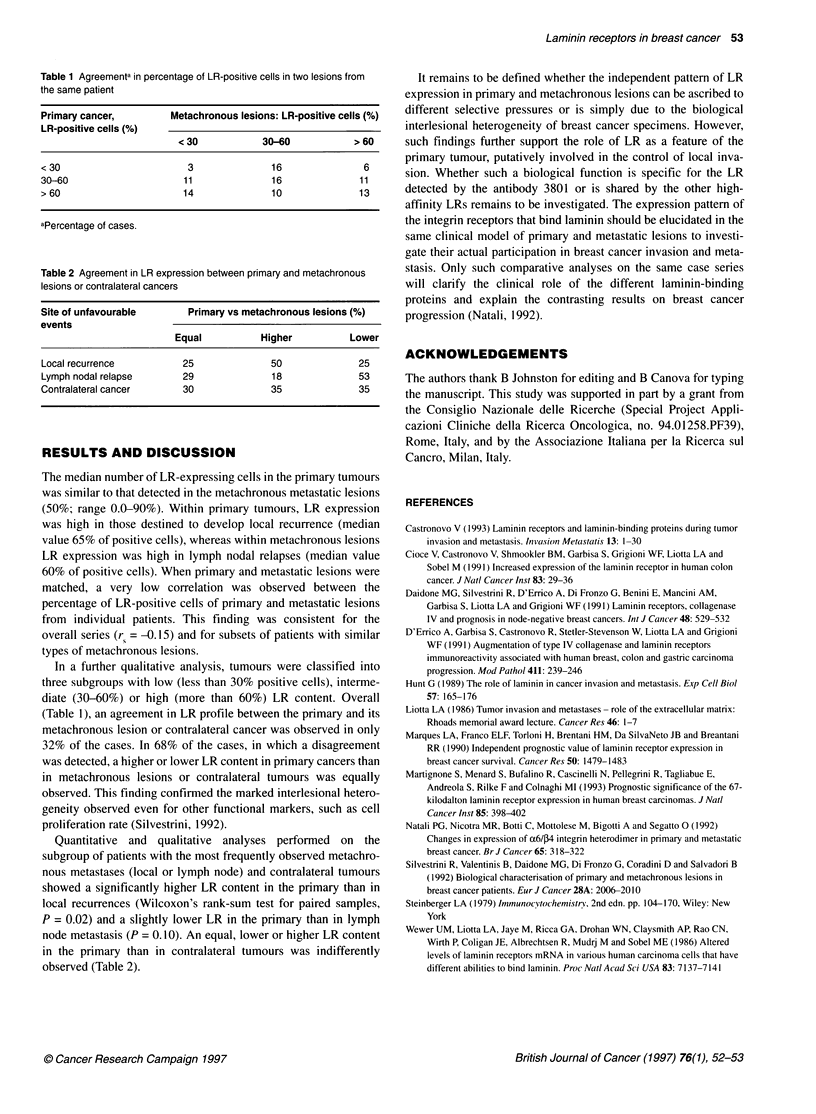# Expression of high-affinity 67-kDa laminin receptors in primary breast cancers and metachronous metastatic lesions or contralateral cancers.

**DOI:** 10.1038/bjc.1997.335

**Published:** 1997

**Authors:** M. G. Daidone, R. Silvestrini, E. Benini, W. F. Grigioni, A. D'Errico

**Affiliations:** Oncologia Sperimentale C, Istituto Nazionale per lo Studio e la Cura del Tumori, Milano, Italy.

## Abstract

The presence of high-affinity 67-kDa laminin receptors, detected immunohistochemically, was determined on 63 primary breast cancers and on metachronous metastatic lesions or contralateral cancers from the same patients. A disagreement was observed in two-thirds of the cases. In particular, laminin receptor content was significantly lower (P = 0.02) in local recurrences and slightly higher in lymph node metastasis than in the corresponding primary tumours.


British Journal of Cancer (1997) 76(1), 52–53
© 1997 Cancer Research Campaign

Short communication

### Expression of high-affinity 67-kDa laminin receptors in primary breast cancers and metachronous metastatic lesions or contralateral cancers

**MG Daidone[1], R Silvestrini[1], E Benini[1], WF Grigioni[2] and A D'Errico[2]**

[1]Oncologia Sperimentale C, Istituto Nazionale per lo Studio e la Cura dei Tumori, Milano; [2]Dipartimento di Patologia, Policlinico S. Orsola, Bologna, Italy

**Summary** The presence of high-affinity 67-kDa laminin receptors, detected immunohistochemically, was determined on 63 primary breast cancers and on metachronous metastatic lesions or contralateral cancers from the same patients. A disagreement was observed in two-thirds of the cases. In particular, laminin receptor content was significantly lower ($P = 0.02$) in local recurrences and slightly higher in lymph node metastasis than in the corresponding primary tumours.

**Keywords:** primary breast cancer; metachronous metastasis; contralateral cancers; laminin receptors

Metastasis is the primary cause of fatal outcome for patients with malignant disease. Metastatic spread includes a cascade of events from tumour cell invasion in the adjacent stroma, migration through the extracellular matrix and invasion into the walls of blood or lymph vessels to colony formation in the secondary sites (Liotta, 1986). Only cells able to adhere to the extracellular matrix component can succeed in metastasis (Liotta, 1986). Interaction between cancer cells and laminin, via expression of laminin receptors (LRs) and/or laminin-binding proteins, plays a critical role during tumour metastasis because laminin is a major component of the basement membrane (Hunt, 1989; Castronovo, 1993). Available information on a large number of laminin-binding proteins with biologically active domains demonstrates the complexity of cellular interactions with laminin.

The identification of several new laminin-binding proteins and the availability of reagents able to detect different LRs belonging to the same gene family have raised the difficult task of attributing specific biological functions, not necessarily involved in invasion and metastasis, to specific receptors and of investigating their clinical role.

Several studies on different tumour types have reported that an increased expression of LR results in a more aggressive phenotype (Cioce et al, 1991; D'Errico et al, 1991; Martignone et al, 1993). However, in breast cancer, such a finding has not been consistently reported (Marques et al, 1990; Daidone et al, 1991), and such contrasting results could be due to the different reagents employed as well as to differences among case series or the clinical end points considered. In particular, in a previous study on a substantial series of patients with node-negative cancers, a high expression of LR was associated with a high frequency of local–regional recurrences but not with other unfavourable events (Daidone et al, 1991). Such a finding could support the hypothesis that activation of the laminin-LR pathway is one of the major factors responsible for local invasion.

In the present study, we determined the expression of LR in primary and metachronous lesions from individual patients also in relation to metastatic site.

*Received 31 May 1996*
*Revised 30 October 1996*
*Accepted 18 November 1996*

*Correspondence to:* R Silvestrini, Oncologia Sperimentale C, Istituto Nazionale Tumori, Via Venezian 1, 20133 Milan, Italy

### PATIENTS AND METHODS

### Tumour material and patient population

LR expression of the primary breast cancer and of its metachronous metastasis was assessed by immunohistochemistry on tumour specimens from 63 patients admitted to the Istituto Nazionale Tumori of Milan during the period September 1978 to April 1989. Determinations on primary tumours were performed at diagnosis before any treatment. The metastatic lesions underwent biopsy for pathological assessment and biological determinations at the time of relapse, which ranged from 6 to 107 months (median 24 months). The metachronous lesions studied were: 24 local recurrences, 17 lymph nodes, five visceral metastases and 17 contralateral cancers. Chemotherapy, hormone therapy or both were given between surgery of the primary tumour and appearance of the metastatic lesion in 38 patients, and 25 patients did not receive any type of systemic treatment.

### Determination of LR

Immediately after surgery or biopsy, tumour material underwent the conventional histological procedure. The presence of LR was evaluated by the immunohistochemical technique previously described (Daidone et al, 1991). From each paraffin-embedded block, 5-µm sections were processed with an anti-LR synthetic peptide-antibody 3801 as previously described (Wewer et al, 1986) using the peroxidase–antiperoxidase method (Steinberger 1979). Negative control slides were treated with preimmune rabbit immunoglobulins instead of specific antibody. Anti-LR antibody stained the cell surface and the cytoplasm. The presence of LR was estimated by two independent observers and was expressed as the percentage of labelled cells over the total number of tumour cells (at least 1000 cells per specimen).

**Table 1** Agreement[a] in percentage of LR-positive cells in two lesions from the same patient

| Primary cancer, LR-positive cells (%) | Metachronous lesions: LR-positive cells (%) | | |
|---|---|---|---|
| | < 30 | 30–60 | > 60 |
| < 30 | 3 | 16 | 6 |
| 30–60 | 11 | 16 | 11 |
| > 60 | 14 | 10 | 13 |

[a]Percentage of cases.

**Table 2** Agreement in LR expression between primary and metachronous lesions or contralateral cancers

| Site of unfavourable events | Primary vs metachronous lesions (%) | | |
|---|---|---|---|
| | Equal | Higher | Lower |
| Local recurrence | 25 | 50 | 25 |
| Lymph nodal relapse | 29 | 18 | 53 |
| Contralateral cancer | 30 | 35 | 35 |

### RESULTS AND DISCUSSION

The median number of LR-expressing cells in the primary tumours was similar to that detected in the metachronous metastatic lesions (50%; range 0.0–90%). Within primary tumours, LR expression was high in those destined to develop local recurrence (median value 65% of positive cells), whereas within metachronous lesions LR expression was high in lymph nodal relapses (median value 60% of positive cells). When primary and metastatic lesions were matched, a very low correlation was observed between the percentage of LR-positive cells of primary and metastatic lesions from individual patients. This finding was consistent for the overall series ($r_s$ = –0.15) and for subsets of patients with similar types of metachronous lesions.

In a further qualitative analysis, tumours were classified into three subgroups with low (less than 30% positive cells), intermediate (30–60%) or high (more than 60%) LR content. Overall (Table 1), an agreement in LR profile between the primary and its metachronous lesion or contralateral cancer was observed in only 32% of the cases. In 68% of the cases, in which a disagreement was detected, a higher or lower LR content in primary cancers than in metachronous lesions or contralateral tumours was equally observed. This finding confirmed the marked interlesional heterogeneity observed even for other functional markers, such as cell proliferation rate (Silvestrini, 1992).

Quantitative and qualitative analyses performed on the subgroup of patients with the most frequently observed metachronous metastases (local or lymph node) and contralateral tumours showed a significantly higher LR content in the primary than in local recurrences (Wilcoxon's rank-sum test for paired samples, $P$ = 0.02) and a slightly lower LR in the primary than in lymph node metastasis ($P$ = 0.10). An equal, lower or higher LR content in the primary than in contralateral tumours was indifferently observed (Table 2).

It remains to be defined whether the independent pattern of LR expression in primary and metachronous lesions can be ascribed to different selective pressures or is simply due to the biological interlesional heterogeneity of breast cancer specimens. However, such findings further support the role of LR as a feature of the primary tumour, putatively involved in the control of local invasion. Whether such a biological function is specific for the LR detected by the antibody 3801 or is shared by the other high-affinity LRs remains to be investigated. The expression pattern of the integrin receptors that bind laminin should be elucidated in the same clinical model of primary and metastatic lesions to investigate their actual participation in breast cancer invasion and metastasis. Only such comparative analyses on the same case series will clarify the clinical role of the different laminin-binding proteins and explain the contrasting results on breast cancer progression (Natali, 1992).

### ACKNOWLEDGEMENTS

The authors thank B Johnston for editing and B Canova for typing the manuscript. This study was supported in part by a grant from the Consiglio Nazionale delle Ricerche (Special Project Applicazioni Cliniche della Ricerca Oncologica, no. 94.01258.PF39), Rome, Italy, and by the Associazione Italiana per la Ricerca sul Cancro, Milan, Italy.

### REFERENCES

Castronovo V (1993) Laminin receptors and laminin-binding proteins during tumor invasion and metastasis. *Invasion Metastatis* **13**: 1–30

Cioce V, Castronovo V, Shmookler BM, Garbisa S, Grigioni WF, Liotta LA and Sobel M (1991) Increased expression of the laminin receptor in human colon cancer. *J Natl Cancer Inst* **83**: 29–36

Daidone MG, Silvestrini R, D'Errico A, Di Fronzo G, Benini E, Mancini AM, Garbisa S, Liotta LA and Grigioni WF (1991) Laminin receptors, collagenase IV and prognosis in node-negative breast cancers. *Int J Cancer* **48**: 529–532

D'Errico A, Garbisa S, Castronovo R, Stetler-Stevenson W, Liotta LA and Grigioni WF (1991) Augmentation of type IV collagenase and laminin receptors immunoreactivity associated with human breast, colon and gastric carcinoma progression. *Mod Pathol* **411**: 239–246

Hunt G (1989) The role of laminin in cancer invasion and metastasis. *Exp Cell Biol* **57**: 165–176

Liotta LA (1986) Tumor invasion and metastases – role of the extracellular matrix: Rhoads memorial award lecture. *Cancer Res* **46**: 1–7

Marques LA, Franco ELF, Torloni H, Brentani HM, Da SilvaNeto JB and Breantani RR (1990) Independent prognostic value of laminin receptor expression in breast cancer survival. *Cancer Res* **50**: 1479–1483

Martignone S, Menard S, Bufalino R, Cascinelli N, Pellegrini R, Tagliabue E, Andreola S, Rilke F and Colnaghi MI (1993) Prognostic significance of the 67-kilodalton laminin receptor expression in human breast carcinomas. *J Natl Cancer Inst* **85**: 398–402

Natali PG, Nicotra MR, Botti C, Mottolese M, Bigotti A and Segatto O (1992) Changes in expression of α6/β4 integrin heterodimer in primary and metastatic breast cancer. *Br J Cancer* **65**: 318–322

Silvestrini R, Valentinis B, Daidone MG, Di Fronzo G, Coradini D and Salvadori B (1992) Biological characterisation of primary and metachronous lesions in breast cancer patients. *Eur J Cancer* **28A**: 2006–2010

Steinberger LA (1979) *Immunocytochemistry*, 2nd edn. pp. 104–170, Wiley: New York

Wewer UM, Liotta LA, Jaye M, Ricca GA, Drohan WN, Claysmith AP, Rao CN, Wirth P, Coligan JE, Albrechtsen R, Mudrj M and Sobel ME (1986) Altered levels of laminin receptors mRNA in various human carcinoma cells that have different abilities to bind laminin. *Proc Natl Acad Sci USA* **83**: 7137–7141

© Cancer Research Campaign 1997